# Comparative Analytical Study of SCMA Detection Methods for PA Nonlinearity Mitigation

**DOI:** 10.3390/s21248408

**Published:** 2021-12-16

**Authors:** Elie Sfeir, Rangeet Mitra, Georges Kaddoum, Vimal Bhatia

**Affiliations:** 1 LaCIME, Génie Electrique, École De Technologie Supérieure, Montreal, QC H3C1K3, Canada; rangeet.mitra.1@ens.etsmtl.ca (R.M.); georges.kaddoum@etsmtl.ca (G.K.); 2 Indian Institute of Technology Indore, Indore 453552, India; vbhatia@iiti.ac.in

**Keywords:** PA nonlinearity, Bussgang-based approach, SCMA, RKHS

## Abstract

Non-orthogonal multiple access (NOMA) has emerged as a promising technology that allows for multiplexing several users over limited time-frequency resources. Among existing NOMA methods, sparse code multiple access (SCMA) is especially attractive; not only for its coding gain using suitable codebook design methodologies, but also for the guarantee of optimal detection using message passing algorithm (MPA). Despite SCMA’s benefits, the bit error rate (BER) performance of SCMA systems is known to degrade due to nonlinear power amplifiers at the transmitter. To mitigate this degradation, two types of detectors have recently emerged, namely, the Bussgang-based approaches and the reproducing kernel Hilbert space (RKHS)-based approaches. This paper presents analytical results on the error-floor of the Bussgang-based MPA, and compares it with a universally optimal RKHS-based MPA using random Fourier features (RFF). Although the Bussgang-based MPA is computationally simpler, it attains a higher BER floor compared to its RKHS-based counterpart. This error floor and the BER’s performance gap are quantified analytically and validated via computer simulations.

## 1. Introduction

Next-generation communication systems must be capable of providing several users/ devices with appropriate service levels for the industrial internet of things (IIoT) and Industry 4.0 [1]. In the context of multiple-access techniques for these ecosystems, non-orthogonal multiple access (NOMA) has emerged as a promising solution that has the potential to support several users over a finite number of temporal/spectral resources. NOMA-based approaches are broadly categorized into the following types [1,2]: (a) power domain NOMA (PD-NOMA), and (b) code domain NOMA. PD-NOMA uses superposition coding to overlap multiple users and detects corresponding user symbols on the receiver side by successive interference cancellation (SIC) or message passing algorithms (MPAs). However, PD-NOMA is known to support a limited number of users due to inter-layer error propagation, and its reliance on power diversity [3,4,5]. Apart from PD-NOMA, specific code-domain NOMA-based approaches, like sparse code multiple access (SCMA) have recently been found to be particularly promising [6,7,8,9], as they not only allow for potential coding/shaping gains through codebook design, but also enable near-optimal detection using MPAs. Besides, SCMA is also known for its robustness to error propagation.

However, transmit-side power amplifier (PA) nonlinearities have been found to degrade the performance of generic SCMA systems. From Bussgang’s theorem [10], transmit-side PA nonlinearity is known to add an independent equivalent distortion noise term that lowers the overall signal-to-noise ratio. Two types of competing MPA-based detection methods exist to mitigate this degradation: (a) Bussgang decomposition-based MPA detectors [11] and (b) random Fourier feature (RFF)-based detectors [12]. While decomposition-based approaches achieve commendable performance under a limited implementation budget, the RFF based approaches offer benefits like universal approximation and generalization across various types of nonlinear PA characteristics. However, RFF-based approaches have slightly more computational overhead, and in certain hardware limited IIoT ecosystems, the implementation complexity of algorithms outweighs the error-floor reached subject to the achievement of a minimum level of quality of service (QoS) [13,14,15]. Therefore, it is compelling to compare and derive analytical insights/comparisons on the error floors of the Bussgang-based MPA methods and to decide on the suitability of a detector for a given bit error rate (BER)-based on the QoS. Several works in the literature have studied the nonlinearity effect not only in SCMA but also in other environments, such as [16], where a Bussgang-based receiver design was proposed for nonlinear PD-NOMA. Moreover, in [12], a nonlinear SCMA system model was studied, and a RFF-based solution was proposed to improve BER performance as equivalent to that obtained in the presence of a linear AWGN channel, whereas an iterative method based on clipping noise was proposed in [11]. Additionally, in [17], RFF-KLMS based algorithm was proposed to mitigate nonlinearity in MIMO-VLC channels.

*Contributions*: In this paper, we present rigorous analytical studies and insights on the optimality of the Bussgang-based MPA for downlink SCMA with PA impairments. From our analysis, the Bussgang-based MPA detector is found to reach a non-negligible BER floor compared to the universally optimal RFF-based MPA, and the analytical results are presented to quantify the BER floor. Next, these results are validated using computer simulations under different fading distributions. The quantification of this error floor could potentially allow for switching between detection methods in hardware-constrained IIoT environments, where meeting a specific QoS constraint with minimal computations is of paramount importance.

## 2. System Model

In this section, we describe the system model considered. We consider a downlink SCMA scenario, in which the users’ bitstreams (considered binary, independent and identically distributed) are grouped and mapped to respective codewords from a codebook ${\left\{x^{\left(j\right)}\in C^{\left(j\right)}\right\}}_{j=1}^{J}$, where each codeword, $x^{\left(j\right)}\in C^{V}$. Furthermore, the number of codewords in each codebook is denoted by $\mathrm{Card}[C^{u}]=M$, with *M* denoting the modulation order, and Card[·] denoting the number of vectors in a codebook. In this paper, we consider a downlink SCMA system as in ([2] Equation (12.3)), where the users’ codewords are overlapped and the superposition, x, is broadcast through the channel h. At the receiver, the received vector, y, is used for MPA-based detection. This is in contrast with the possible uplink scenario presented in ([2] Equation (12.1)) where the users’ codewords could arrive asynchronously. For this hypothetical case, there is indeed a possibility of interference between the codewords that could impair their sparsity/algebraic-structure; however, this issue does not arise for downlink SCMA.

For *V* non-interfering resources, the observation at the receiver, $y\in C^{V}$, is given as [2], ([12] Equation (12.3)):

(1) $$y=\mathrm{diag}\left(h\right)f\left(\underset{X}{\underset{︸}{\sum_{j=1}^{J}x^{\left(j\right)}}}\right)+n,$$

where f(·) denotes the PA nonlinearity, x denotes the instantaneous superposition of the users’ codewords, diag(·) is a diagonal matrix that contains elements of (·) in its diagonal, and $h\in C^{V}$ is a vector of channel gains sampled according to a probability density function (PDF) p(h). The contribution in this work is not constrained by prior statistical assumptions on h. Furthermore, the complex additive white Gaussian noise (AWGN) vector is given by $n={\left[n_{1},n_{2},\cdots ,n_{V}\right]}^{T}$, with each $n_{i}∼p\left(n\right)$. Without sacrificing generality, we consider AM-AM Rapp nonlinearity for the PA model, f(x), which is expressed as follows [18]:

(2) $$\begin{array}{c} f\left(x\right)=\frac{x}{{\left(1+{\left|\frac{x}{x_{\mathrm{sat}}}\right|}^{2p}\right)}^{\frac{1}{2p}}}, \end{array}$$

where *p* denotes the parameter that controls the severity of the nonlinearity, and $x_{sat}$ is the PA saturation voltage. It is noted that the RFF-based detectors’ performance is not dependent on the nonlinear PA characteristics or their knowledge at the receiver, and existing works show their generalization across different PA characteristics [12].

**Figure 1:**
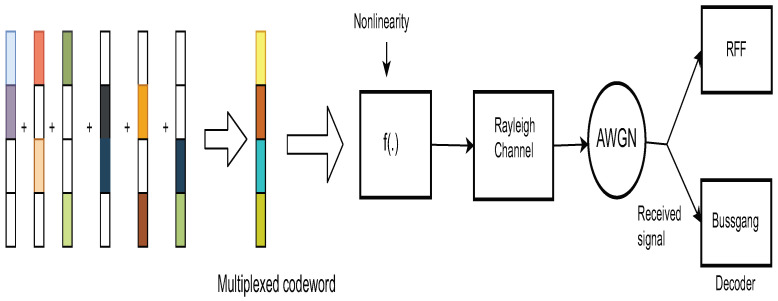
Depiction of the System Model for SCMA.

**Figure 2:**
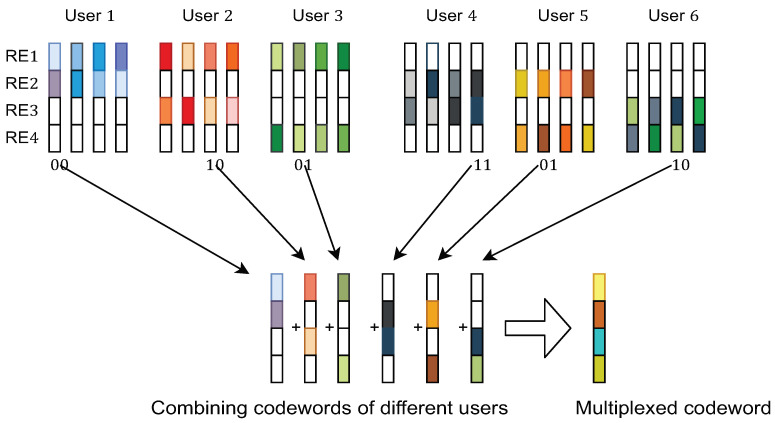
Depiction of the overlapping of codewords for different users.

**Figure 3:**
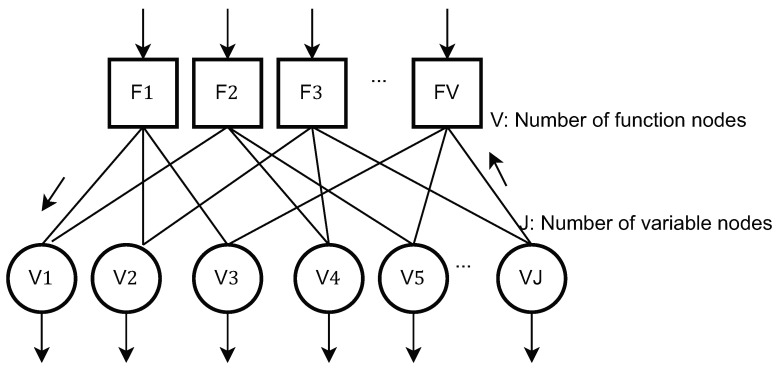
Depiction of the Tanner Graph.

The components of the system model are pictorially depicted in Figure 1, Figure 2 and Figure 3. The transmitter model described mathematically in (1) is shown pictorially in Figure 1. Figure 2 pictorially depicts the overlap of the codewords from each users’ dictionary. Finally, the dependence on the user-resources and the variable-nodes is shown by a Tanner graph in Figure 3.

## 3. Bussgang Decomposition-Based MPA

In this section, we elaborate on the Bussgang decomposition-based MPA detector. The MPA detector iteratively exchanges the log(·) of the conditional likelihood as messages across the function nodes, indexed as j=1,2,⋯,V, and the variable nodes, indexed as k=1,2,⋯,J. Also, for the resulting Tanner graph of the function nodes and variable nodes ([2] Section 12.1.1.3), the graph neighborhood of node *k* is denoted as $B_{k}$. In this regard, we invoke the Bussgang theorem [10], and re-express (1) as:

(3) y=αdiag(h)x+v+n,

where α denotes a correlation-coefficient and v denotes an independent distortion term with variance $\sigma_{v}^{2}$. Using this equivalent form, we obtain the following expression for the conditional PDF, p(y[k]|x):

(4) $$\begin{array}{c} p\left(y\left[k\right]|x\right)=\frac{1}{2\pi \sigma_{n}^{2}}\exp\left[-\frac{{\left|y\left[k\right]-\alpha h\left[k\right]\sum_{\forall j\in B_{k}}x\left[k\right]\right|}^{2}}{\sigma_{n}^{2}+\sigma_{v}^{2}}\right]. \end{array}$$

Generally, classical MPA-based detection propagates the log(·) of the conditional PDF across the function nodes, *j*, and variable nodes, *k* ([2] p. 377). For AWGN channels, the conditional PDF of y[k] given x is provided below:

(5) $$\begin{array}{c} \log p\left(y\left[k\right]|x\right)=-\log\left(2\pi \sigma_{n}^{2}\right)-\frac{{\left|y\left[k\right]-\alpha h\left[k\right]\sum_{\forall j\in B_{k}}x\left[k\right]\right|}^{2}}{\sigma_{v}^{2}+\sigma_{n}^{2}}. \end{array}$$

The parameters α and $\sigma_{v}^{2}$ are estimated using the available pilots and the channel estimates h from (1) as follows:

(6) $$\begin{array}{c} \alpha =\frac{E\left[y^{T}\mathrm{diag}\left(h\right)x\right]}{E\left[{∥\mathrm{diag}(h)x∥}^{2}\right]},\\ \sigma_{v}^{2}={\left(1-\alpha \right)}^{2}E\left[{∥\mathrm{diag}(h)x∥}^{2}\right]. \end{array}$$

For the log-max MPA approaches over AWGN channels, the messages, $m_{jk}$, are essentially given by the log likelihood $\log\left[p(y[k]|x)\right]$. Considering the Bussgang representation of (1) in (3), $m_{jk}$ is explicitly written as:

(7) $$m_{jk}=\frac{{-\left|y\left[k\right]-\alpha h\left[k\right]\sum_{\forall j\in B_{k}}x\left[k\right]\right|}^{2}}{\sigma_{n}^{2}+\sigma_{v}^{2}}.$$

The difference between the value of this message and its corresponding ideal value is expressed as follows:

(8) $$\Delta m_{jk}=\frac{{\underset{︸}{{\left|y\left[k\right]-h\left[k\right]\sum_{\forall j\in B_{k}}x\left[k\right]\right|}^{2}\left(\sigma_{n}^{2}+\sigma_{v}^{2}\right)}}_{P}-{\underset{︸}{{\left|y\left[k\right]-\alpha h\left[k\right]\sum_{\forall j\in B_{k}}x\left[k\right]\right|}^{2}\sigma_{n}^{2}}}_{Q}}{\sigma_{n}^{2}\left(\sigma_{n}^{2}+\sigma_{v}^{2}\right)}.$$

If the appropriate expression for the Kullback–Leibler divergence between Gaussian PDFs having zero mean and variances $\sigma_{n}^{2}$ and $\sigma_{n}^{2}+\sigma_{d}^{2}$ is invoked, the difference between $m_{jk}$ and its corresponding ideal value, $E\left[\Delta m_{jk}\right]$ (with α=1 and $\sigma_{v}^{2}=0$), is given by [19]:

(9) $$E\left[\Delta m_{jk}\right]=\frac{1}{2}\log\left[\frac{\sigma_{n}^{2}+{\left(1-\alpha \right)}^{2}\sigma_{h}^{2}\sigma_{x}^{2}+\sigma_{n}^{2}}{\sigma_{n}^{2}}\right]+\frac{\sigma_{n}^{2}}{2\left(\sigma_{n}^{2}+{\left(1-\alpha \right)}^{2}\sigma_{h}^{2}\sigma_{x}^{2}\right)}-\frac{1}{2},$$

where

(10) $$\begin{array}{c} \sigma_{h}^{2}=E\left[h^{2}\left[k\right]\right],\\ \sigma_{x}^{2}=E\left[{\left(\sum_{\forall j\in B_{k}}x\left[k\right]\right)}^{2}\right]. \end{array}$$

Next, we directly link the converged log likelihood ratio for the ideal linear channel to the generalized signal-to-noise ratio (GSNR) [20], ref. [21] achieved at convergence, SNR*, which is in turn a function of $\psi_{p\left(h\right)}$ (the PDF of the channel gain) [20]:

(11) $${\mathrm{BER}}_{\mathrm{Linear}}=\psi_{p\left(h\right)}\left({\mathrm{SNR}}^{*}\right).$$

From the expression for the message error derived in (9), the BER of the proposed Bussgang detector, ${\mathrm{BER}}_{\mathrm{Bussgang}}$, is approximately expressed as:

(12) $${\mathrm{BER}}_{\mathrm{Bussgang}}=\psi_{p\left(h\right)}\left({\mathrm{SNR}}^{*}\right)+\psi_{p\left(h\right)}^{\prime }\left({\mathrm{SNR}}^{*}\right)\times E\left[\Delta m_{jk}\right],$$

where the $E\left[\Delta m_{jk}\right]$ is derived in (9). The following insights are drawn from the above analytical result:

- Notably, (12) quantifies the gap between the BER of the proposed approach and that of a universally optimal MPA (the RFF-based MPA in [12]). As mentioned before, this quantification helps when trading off computational complexity with BER performance subject to achieving a given BER-based level of QoS.
- It is further noted that the above deviation is independent of the fading distribution. In this context, it is indeed worth mentioning that the ideal BER, $\psi_{p(h)}\left({\mathrm{SNR}}^{*}\right)$, is mostly an integral of a Q-function over the concerned PDF p(h) [2]. However, when $\psi_{p(h)}\left({\mathrm{SNR}}^{*}\right)$ (and hence its derivative $\psi_{p\left(h\right)}^{\prime }$) are known, the optimality gap is found to be independent of the underlying distribution.
- It is possible to further improve the error approximation in (12) as follows:
  (13) $${\mathrm{BER}}_{\mathrm{Bussgang}}=\sum_{l=0}^{\infty }\frac{\psi_{p(h)}^{\left(l\right)}\left({\mathrm{SNR}}^{*}\right)}{l!}E\left[\Delta m_{jk}^{l}\right],$$
  where $\psi_{p(h)}^{\left(l\right)}\left(\cdot \right)$ represents the $l^{th}$ derivative of $\psi_{p(h)}\left(\cdot \right)$. To simplify, we note from (7) that $P,Q∼\mathrm{Exp}\left[\sigma_{n}^{2}\left(\sigma_{n}^{2}+\sigma_{v}^{2}\right)\right]$ are even powers of normal random variables with average energy $\sigma_{n}^{2}\left(\sigma_{n}^{2}+\sigma_{v}^{2}\right)$. Therefore, we obtain the following for $E\left[\Delta m_{jk}^{l}\right]$:
  (14) EΔmjkl=∑s=0llsEPsQl−s.
  From ([22] p. 546), this is simplified as:
  (15) EΔmjkl=∑s=0l∑u=0mins,(l−s)ls2s!2l−s!ασh2σx22u2l(s−u)!(l−s−u)!2u!,
  which yields the final expression:
  (16) $${\mathrm{BER}}_{\mathrm{Bussgang}}=\sum_{l=0}^{\infty }\frac{\psi_{p(h)}^{\left(l\right)}\left({\mathrm{SNR}}^{*}\right)}{l!}E\left[\Delta m_{jk}^{l}\right].$$

A summary of the proposed Bussgang-based MPA is provided in Algorithm 1.


**Algorithm 1** Bussgang based MPA.
1:   Initialization:
     $I_{kj}=p\left(x_{j}\right)$ according to a uniform distribution.
2:   Initialization:
     $I_{jk}:=\frac{1}{2\pi \sigma_{n}^{2}}\exp\left[-\frac{{\left|y\left[k\right]-\alpha h\left[k\right]\sum_{\forall j\in B_{k}}x\left[k\right]\right|}^{2}}{\sigma_{v}^{2}+\sigma_{n}^{2}}\right]$
     $\alpha =\frac{E\left[y^{T}\mathrm{diag}\left(h\right)x\right]}{E\left[{∥\mathrm{diag}(h)x∥}^{2}\right]}$,
     $\sigma_{v}^{2}={\left(1-\alpha \right)}^{2}E\left[{∥\mathrm{diag}(h)x∥}^{2}\right]$.
3:   Initialize the maximum number of iterations, ITER.
4:   **while** *c* < *ITER* **do**
     $I_{jk}:=\log\left(p\left(x_{j}\right)\right)+\sum_{j\in B_{k}}I_{kj}.$
     $I_{kj}:=\max_{\forall x_{j}\in C_{j},k\in B_{j}}\log\left(p\left(y\left[k\right]|x\right)\right)+\sum_{k\in B_{j}}I_{jk}$
     c:=c+1
   **end while**
5:   Detect user-symbols as per ([2] Equation (12.12)) using the steady-state message-values $I_{jk}$ and codebook $C_{j}$



## 4. Simulations

**Figure 4:**
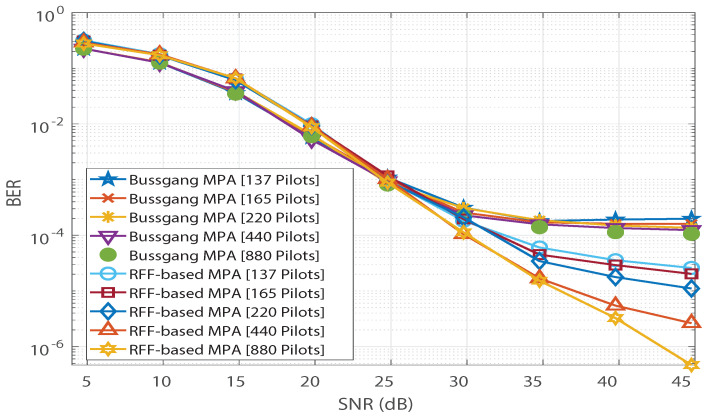
BER vs. SNR comparison of the Bussgang-based detector with RFF-based detector for a Rayleigh Channel by varying the number of pilots.

In this section, we present the simulation results to validate the Bussgang decomposition-based MPA. Without sacrificing generality, a simplistic codebook from [23] is considered in our simulations. We set p=1 and $x_{sat}$ to be equal to the maximum dynamic range of x. Furthermore, the BER simulations are performed over $10^{7}$ bits, and 15 MPA iterations are used. The simulation results for a Rayleigh channel are depicted in Figure 4. The simulation parameters are summarized in Table 1.

**Figure 5:**
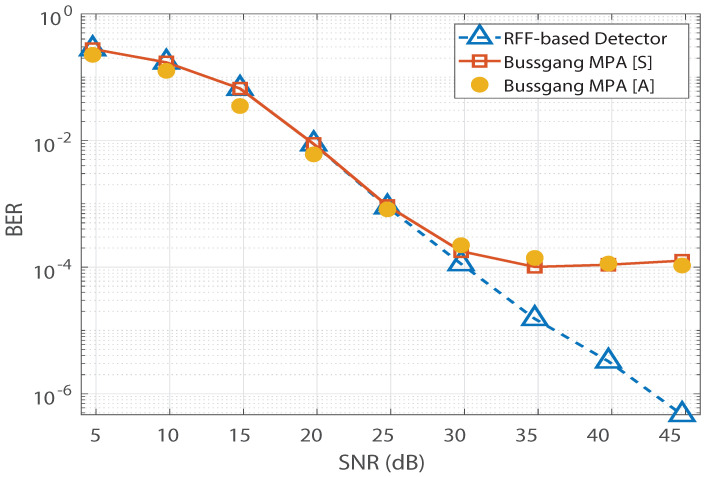
BER vs. SNR validation for the Bussgang-based detector for a Rayleigh channel.

**Figure 6:**
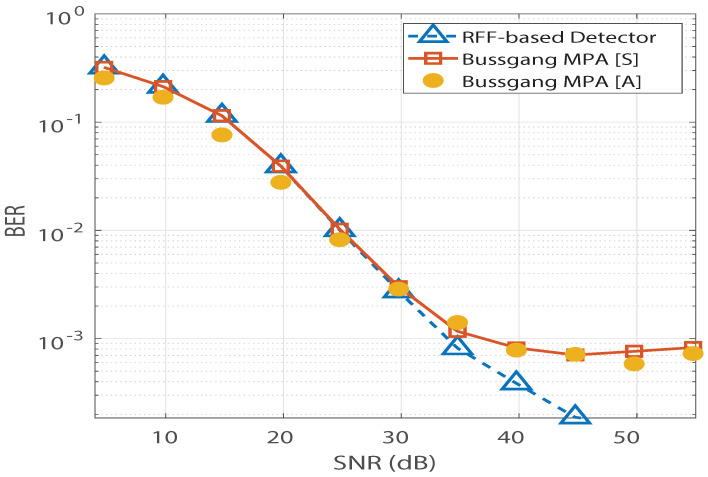
BER vs. SNR validation for the Bussgang-based detector for a Nakagami-*m* channel with m=0.5.

In Figure 4, saturation is observed in Bussgang-based MPA’s BER performance. In addition, we observe no significant change in Bussgang-based MPA’s BER floor when the number of pilots is increased from 137 to 880. However, for the RFF-based MPA detection in [12], its BER performance is found to improve as the number of pilots increases, and the saturation due to the BER floor is completely invisible at 880 pilots. Furthermore, the analytical expression for the BER of the Bussgang-based detector derived in (12) is validated in Figure 5, which illustrates close agreement between the analytical BER (denoted by [A]) and the simulated BER (denoted by [S]). Figure 6 shows a similar validation of the analytical result derived in (12) assuming a Nakagami-*m* distributed h, with m=0.5. Since the mode of the Nakagami-*m* distribution (with m=0.5) is zero, we observe degraded BER performance for Nakagami-*m* fading as compared to the BER performance for the Rayleigh channel presented in Figure 5. However, due to the distribution-independent quantification of the performance gap presented in (12), a close match is observed between the simulated BER and the analytical BER for the Bussgang-based detector in Figure 6. This quantification of the BER floor helps when predetermining the viability of using a lightweight Bussgang-based MPA (which has a complexity of $O(TKM^{d_{f}})$, where $d_{f}$ denotes the free distance) over a complex RFF-based detector (which has a complexity of $O(TKM^{d_{f}}+n_{G}^{2})$, where $n_{G}$ denotes the number of RFFs) subject to achieving a BER-based level of QoS.

## 5. Conclusions

In this paper, a low-complexity detector, the Bussgang-based MPA, was derived, and its BER performance was quantified. The proposed detector was found to present a BER floor comparable to that of existing RFF-based approaches. The BER floor was quantified analytically relative to the optimal RFF-based MPA without specific assumptions about the nature of the PA nonlinearity or the fading distribution. Additionally, the analytical results were validated by computer simulations considering different channel distributions. The detector is attractive despite its error floor due to its simplicity and suitability for hardware-limited IIoT systems, wherein achieving a certain level of QoS with low computational cost outweighs the requirement of obtaining a universally optimal BER performance.

## Figures and Tables

**Table 1:** Simulation Parameters.

Codebook	Section II.A [23]
Modulation	OOK
Value of p	1
Kernel-width assignment	Silverman’s rule [24]
Number of MPA iterations	15
Number of transmitted bits	$10^{7}$
Parameter values for Rayleigh distribution	$\sigma_{h}^{2}=1$
Parameter values for the Nakagami-*m* distribution	Shape: m=0.5,
	Spread parameter: 1
$n_{G}$	110

## Data Availability

Not Applicable.

## Abbreviations

The following abbreviations are used in this manuscript:

NOMA	Non-orthogonal multiple access
SCMA	Sparse code multiple access
MPA	Message passing algorithm
BER	Bit error rate
RKHS	Reproducing kernel Hilbert space
RFF	Random Fourier features
IIoT	Industrial internet of things
PD-NOMA	Power domain NOMA
SIC	Successive interference cancellation
PA	Power amplifier
QoS	Quality of service
PDF	Probability density function
AWGN	Additive white Gaussian noise
GSNR	Generalized signal-to-noise ratio
